# Similar costs and outcomes for differentiated service delivery models for HIV treatment in Uganda

**DOI:** 10.1186/s12913-022-08629-4

**Published:** 2022-11-03

**Authors:** Teresa Guthrie, Charlotte Muheki, Sydney Rosen, Shiba Kanoowe, Stephen Lagony, Ross Greener, Jacqueline Miot, Hudson Balidawa, Josen Kiggundu, Jacqueline Calnan, Seyoum Dejene, Thembi Xulu, Ntombi Sigwebela, Lawrence C Long

**Affiliations:** 1 Health Economics and Epidemiology Research Office, Department of Internal Medicine, School of Clinical Medicine, Faculty of Health Sciences, University of the Witwatersrand, Johannesburg, South Africa; 2 Health Net Consult, Kampala, Uganda; 3 Department of Global Health, School of Public Health, Boston University, Boston, MA United States of America; 4 Ministry of Health, Kampala, Uganda; 5 USAID, Kampala, Uganda; 6 Right To Care, EQUIP, North Centurion, South Africa

**Keywords:** Cost, Health outcomes, Economic evaluation, Uganda, HIV, Differentiated service delivery

## Abstract

This cost-outcome study estimated, from the perspective of the service provider, the total annual cost per client on antiretroviral therapy (ART) and total annual cost per client virally suppressed (defined as < 1000 copies/ml at the time of the study) in Uganda in five ART differentiated service delivery models (DSDMs). These included both facility- and community-based models and the standard of care (SOC), known as the facility-based individual management (FBIM) model. The Ministry of Health (MOH) adopted guidelines for DSDMs in 2017 and sought to measure their costs and outcomes, in order to effectively plan for their resourcing, implementation, and scale-up. In Uganda, the standard of care (FBIM) is considered as a DSDM option for clients requiring specialized treatment and support, or for those who select not to join an alternative DSDM. Note that clients on second-line regimes and considered as “established on treatment” can join a suitable DSDM.

Using retrospective client record review of a cohort of clients over a two-year period, with bottom-up collection of clients’ resource utilization data, top-down collection of above-delivery level and delivery-level providers’ fixed operational costs, and local unit costs. Forty-seven DSDMs located at facilities or community-based points in the four regions of Uganda were included in the study, with 653 adults on ART (> 18 years old) enrolled in a DSDM. The study found that retention in care was 98% for the sample as a whole [96–100%], and viral suppression, 91% [86-93%]. The mean cost to the provider (MOH or NGO implementers) was $152 per annum per client treated, ranging from $141 to $166. Differences among the models’ costs were largely due to clients’ ARV regimens and the proportions of clients on second line regimens. Service delivery costs, excluding ARVs, other medicines and laboratory tests, were modest, ranging from $9.66–16.43 per client per year. We conclude that differentiated ART service delivery in Uganda achieved excellent treatment outcomes at a cost similar to the standard of care. While large budgetary savings might not be immediately realized, the reallocation of “saved” staff time could improve health system efficiency and with their equivalent or better outcomes and large benefits to clients, client-centred differentiated models would nevertheless add great societal value.

**Supplementary Information:**

The online version contains supplementary material available at 10.1186/s12913-022-08629-4.

## Introduction

In 2019, there were an estimated 1.5 million adults living with HIV (PLHIV) in Uganda, equivalent to an HIV adult prevalence of 5.8% [5.4–6.2%] [1]. Approximately 84% of the HIV-positive population were reported to be on antiretroviral therapy (ART), and 90% of those were estimated to be virally suppressed. For Uganda, as for other high HIV prevalence countries, achieving the global 95-95-95 targets will require adapting service delivery approaches to the needs and preferences of PLHIV, with the goal of maintaining good clinical outcomes, reducing costs to clients, and improving efficiency in service delivery [2].

The Ugandan Ministry of Health (MOH) began piloting and scaling-up “differentiated ART service delivery models” (DSDMs) in 2016, becoming one of the first sub-Saharan African countries to develop and implement a comprehensive DSDM program. National guidelines for DSDMs were issued in 2017. Soon after, as uptake of DSDM models expanded, the MOH requested information on their costs and outcomes, in order to plan for the resourcing, implementation, and scale-up of the guidelines.

As of 2018, there were five officially approved DSDMs in Uganda for both established on treatment clients (stable) and complex clients (Table 1): facility-based individual management (FBIM), which is the standard of care (SOC); facility-based groups (FBG); fast-track drug refills (FDR); community client-led ART delivery (CCLAD); and community drug distribution points (CDDP). By December 2020, roughly 79% of all adult ART clients had been enrolled in one of the five models: 42% in FDR, 34% in FBIM, 12% in FBGs, 7% in CCLAD, and 5% in CDDP [3]. The remaining 21% of clients were not recorded as being enrolled in a DSDM and are assumed to have been receiving standard of care treatment at facilities. Importantly, clients on second-line ARV regimens were able to join DSDMs, if they were considered as ‘established on treatment’.

There have been a few prior evaluations of the clinical outcomes of early versions of DSDMs in Uganda [4– 8], but there is little program-wide evidence on costs and effectiveness, a dearth that hinders national budgeting, resource mobilization, implementation planning, and scale-up. At the request of the Ugandan MOH, the United States Presidential Emergency Plan for AIDS Relief (PEPFAR)-funded EQUIP Health Project (a consortium of African organisations focused on improving HIV treatment access in a number of African countries) conducted a cost-outcome analysis, from the perspective of the service provider, of the five DSDMs to estimate the annual cost per person retained in care and per client virally suppressed in each model.

**Table 1:** Differentiated ART service delivery models in Uganda

Ugandan DSDM	Description and clients	When	Where	Who	What
Facility-based individual management (FBIM)	FBIM is the conventional standard-of-care model of ART delivery for clients needing extra attention, such as complex clients, those who have recently been initiated in care, and those who chose to continue to receive their services at the facility.	Mostly monthly or 2-monthly ARV refills and clinical consultations	Primary health care facility	Nurse Pharmacist	ART refills Clinical monitoring Adherence support Laboratory tests OI treatment
Facility-based groups (FBG)	FBGs are for established on treatment or complex clients needing peer support, such as adolescents, pregnant and breastfeeding women (PBFW), and discordant couples.	The frequency of their ARV refills depends on clients’ stability.	Community or Primary health care facility	Lay provider Peers	Adherence support Psychosocial support
Fast-track drug refills (FDR)	Clients who are established on treatment can pick-up their ARVs directly from clinics / pharmacies without clinical consultations (which should be done annually). FDRs can include clients on second-line regimens.	Every 2, 3 or 6 months	Public pharmacy either at primary health facility or hospital	Pharmacist	ART refills
Community client-led ART delivery (CCLAD)	Clients established on treatment form groups within their communities. One person is selected (on rotational basis) to collect the ARV refills for the whole group from the facility.	Monthly, or every 2 or 3 months	Community	Lay provider Peers Nurse (for viral load testing in community)	ART refills Clinical monitoring Adherence support Psychosocial support Viral load testing can be done in community
Community drug distribution points (CDDP)	Clients established on treatment pick up their ARVs from a community outreach point, including private pharmacies	Every 2, 3 or 6 months	Community Or private pharmacy in community	Lay provider Peers Pharmacist	ART refills

Source: [9]

## Methods

In this study, we estimated the annual cost per client outcome of a cohort of Ugandan ART clients enrolled in the five official DSDMs (including the SOC FBIM) in 2017. The cost to providers for individual client resource use was estimated using a bottom up, micro costing approach, with retrospective data drawn from clients’ medical records using methods previously described [10–12]. Public and non-public (private not-for-profit) providers’ and implementing partners’ fixed and shared operational, management, and supervisory costs were estimated using a top-down approach. We compare two periods of observation (study periods): 0–12 months after study enrolment, which corresponds to calendar year 2017 (1 January 2017-31 December 2017), and 13–24 months after study enrolment, which corresponds to calendar year 2018 (1 January 2018-31 December 2018). These are study observation periods only; they do not refer to clients’ duration on ART or time in the DSDM. Costs are reported from the provider’s perspective only.

### Study sites

Study sites were selected to capture the variation in settings, implementing partners, and other characteristics of ART services in Uganda. We define a “site-model” as one model being implemented by one ART facility, though model services may be delivered at non-facility locations. Using this definition, a facility can have more than one site-model if more than one differentiated model is offered there. Our sampling frame included any site-model which had been in operation for ≥ 6 months by January 2017. Site-models that were considered outliers in terms of size (number of clients) or access (extreme locations that were physically difficult to reach) were excluded from the sampling frame. In January 2017, there were 605 site-models that met our sample criteria at 297 facilities in Uganda. From these, multi-stage purposive sampling was used to select 47 site-models so as to reflect variation according to model type, facility ownership (public and private-not-for-profit), client volume, geographic location, and implementing partner (further details on the sampling criteria are included in Supporting information S1 File). We note that many of the public facilities in the study were supported by a non-governmental “implementing partner” receiving external donor support largely from PEPFAR. These implementers played a major role in establishing and maintaining the DSDMs. We thus captured their operational costs, as well as those of the MOH.

Fixed costs for providers and implementers were collected for all 47 site-models. Twenty of the 47 (4 per DSDM) were then selected for the collection of client level resource usage and treatment outcomes.

### Study population

All adult ART clients (≥ 18 years) who were enrolled in a DSDM on or before 1 January 2017 were eligible for our study. We did not include adolescents and children in our sample due to the complexities in obtaining ethical permission to access their records and groups, as well as practical limitations in accessing their DSDM services. In Uganda, all PLHIV are eligible for DSDMs, *irrespective of their ARV regimen (first or second-line)*, but their specific model options depend on model availability and clinical stability. A client who is considered established on treatment is defined as one who is (a) on their current ART regimen (not necessarily first-line regimens only) for ≥ 12 months; (b) virally suppressed; (c) in WHO Stage I/II; (d) adherent (> 95%) over the last 6 months; and (e) if a TB client, past the intensive TB treatment phase (2 months) and sputum negative. The proportion of sampled clients on second-line regimens in each DSDM affected the average cost per client due to greater ARV prices. Clients who met these eligibility criteria were selected consecutively from DSDM registers kept by the facilities starting in January 2017 and then sequentially earlier in time (December 2016, November 2016, and so on) until the target sample size of 30–33 clients was reached for each of the 20 sites. Clients with a record of formal transfer out of a selected health facility before the 12-month study endpoint were excluded. For the FBG sites, only groups for pregnant and breastfeeding women (PBFW) were selected because of more rigorous ethical clearance requirements for accessing pediatric and youth groups and the small number of sero-discordant couple groups.

Participants in each of the models except FBGs were followed longitudinally for 24 months starting on January 1, 2017. This follow-up period was broken into two periods: 0–12 months after study enrolment and 13–24 months after study enrolment, with data accessed retrospectively at the end of each period. For the FBGs, two different samples of PBFW were followed for each 12 month period (FBG1 and FBG2) because they only remained in their FBG for the duration of their pregnancy and postnatal period.

### Data collection

All data for the study were collected locally from three sources. First, research assistants retrospectively extracted demographic characteristics, dates of and reasons for clinic visits, laboratory tests, counselling sessions, ARVs and non-ARV medications dispensed, TB status, WHO clinical stage and treatment outcomes from study participants’ ART care cards, which were maintained by facility staff. A number of participants presented with comorbidities and other illnesses, however these may have been inconsistently recorded on their ART cards, and therefore could not be reported in a systematic way. Second, from model-specific DSDM registers, participants’ attendance at any DSDM-related event were recorded (adherence counselling, group support meetings, FBG meetings, community medication collection/distribution meetings, viral load counselling and testing (drawing bloods) sessions conducted in the community etc.). Third, we interviewed programme and financial managers at each of the site-models, collected the estimated length of time spent by the different cadre for each service, obtained expenditure records, asset registers and undertook spacial measurements of the buildings used in providing the DSDM services.

### Treatment outcome measures

Retention in care and viral suppression rates as reported in individual participants’ medical records were the primary treatment outcomes of interest in this analysis. At the time of the commencement of the study, ‘retention’ was measured as not having missed a scheduled appointment (clinic or DSDM) for > 90 consecutive days, and viral suppression was based on the latest viral load (VL) test in each study period (12/24 month ± 3-month window) being < 1000 copies/ml, both definitions consistent with Uganda MOH practice at the time of the study. The definition of retention has subsequently been adjusted to only 28 days, which would affect the rates of retention observed in our study sample. For the cost analysis, we defined four mutually exclusive outcomes as follows: Retained in care and known to be virally suppressed (RIC, suppressed); retained in care and known to be not virally suppressed (RIC, unsuppressed); retained in care and VL unknown (RIC, VL unknown); not in care (NIC). ARV adherence, as proxied by an annual medication possession ratio (MPR) (total days dispensed/365), was reported as a secondary treatment outcome and categorized using the MOH’s scale (good ≥ 95%; fair 85-94%; poor 75-84%; non-adherent ≤ 74%). Clients in the cohort who switched between models during the study period were retained in their original models for analysis.

### Resource utilization, cost data and cost analysis

To calculate direct resource utilization for each client, we identified and quantified all resources utilized within the two 12-month study periods. Client-level resource utilization data were identified and quantified from clients’ ART care cards and DSDM registers, as described above.

The cost per unit of each resource were collected from price lists, salary scales, tender documents, and implementers’ expenditure logs. Staff costs per facility visit or DSDM event were calculated based on the estimated time per visit or DSDM event for each staff member at the average cost of that cadre’s time, based on total remuneration. The estimated time per visit was estimated from staff interviews. Quantities of resources used were multiplied by unit costs and summed to obtain an average direct cost per client. (Details of prices and costing methods are described in Supporting information S1 Table and S2 Table).

We also estimated indirect (fixed and shared) costs, including facility and DSDM management, administration, oversight and supervision, staff training, equipment, building/ rental and all operational and overhead costs at the facility and above-facility levels. These indirect costs, varying by model type, were attributed to each DSDM client using an allocation factor based on facility annual headcount (out-client visits) and each client’s number of visits.

Finally, we summed the direct and indirect cost/client to generate a total cost per client, stratified by DSDM-type and client outcome. Cost estimates were presented with the appropriate summary statistics (mean and standard deviation). We also estimated the “production cost” of achieving one client who was virally suppressed by dividing the total cost (any outcome) per model by the proportion of clients with viral suppression in that model.

Unit costs reflect 2018 market prices and were converted from Uganda shillings to United States dollars (USD) using the annual average Bank of Uganda exchange rate for 2018 of $1:UGX 3728 [13]. Costs are reported in 2018 USD.

## Results

### Study population

A total of 653 clients from four regions of Uganda (Central, East, North and West) were enrolled in the study, divided roughly evenly among the five DSDM types (Table 2). During the two-year study period, 29 clients switched back to FBIM due to viral failure, while 6 FBIM clients switched to other DSDMs. As explained above, these clients were retained in their original models for purposes of analysis.

The majority (473, 72%) were female, a slightly higher proportion than in the national ART cohort due to our sampled FBG participants being all female. The facility-based individual models (FDR and FBIM) had the highest proportions of male participants: 58 (44%) and 46 (36%), respectively. The median age for all the models except FBG ranged from 41 to 44 years; FBG clients were younger, with a median age of 29 years. The median duration on ART was 5 years; FBIM and FBG clients had been on ART for less time (2 and 3 years respectively) and had the highest median baseline CD4 counts (310 and 433 cells/µl respectively), while FDR clients had spent a median of 8 years on ART. At study enrolment, the median length of time in a differentiated model was one year, and 593 (91%) of clients were on first line (FL) ARV regimens. Only the FDR model cohort reported more than 10% of participants on a second line (SL) regimen (22, 17%), the FBIM sample had the next highest share of clients on SL regimens (11, 9%). Unfortunately, no indication was given on the clients’ care cards as to if and why FBIM clients opted to remain in the SOC option, rather than other DSDM options. It is also important to note that in the early years of the DSDMs (when this study was undertaken), nurses tended to select clients who they judged to be “more established’ on treatment to join the new DSDMs (Table 2).

### Treatment outcomes

Overall retention in care and viral suppression rates were high for all the models (Table 2). For the sample as a whole, retention in care was 97% and 98% at 12 and 24 months, respectively; average viral suppression was 91% for both periods. FBIM clients had the highest proportion of known non-suppressed clients (9.4% and 7.9%) and was the only model to report a death, which occurred in the second study period, while FBG had the highest suppression rate at 94%. The majority of clients in both study periods (80% and 83%, respectively) were classified as having “good” adherence (≥ 95%), based on the annual MPR and the scale provided by the MOH (where the FBIM mean ARV days prescribed for the year were 364, CCLAD 361, CDDP 363, FBG 364 and FDR 369).

**Table 2:** Characteristics and treatment outcomes by model of ART delivery

Sample characteristics (n, % unless otherwise specified)	FBIM (n = 128)	CCLAD (n = 131)	CDDP (n = 132)	FBG1 /2 (n = 129, 115)^a^	FDR (n = 133)	Total (n = 653)
Sex (female)	82 (64%)	92 (70%)	95 (72%)	129 (100%)	75 (56%)	473 (72%)
Age, years (median, IQR)*	41 (34–51)	44 (40–49)	44 (38–52)	29 (25–34)	44 (35–51)	41 (33–48)
Duration on ART, years (median, IQR)*	3 (2–5)	5 (2–8)	7 (5–10)	2 (1–3)	8 (5–10)	5 (2–8)
Duration in DSDM, years (median, IQR)^b^	3 (2–5)^c^	1 (1–1)	1 (1–6)	1 (1–1)	2 (1–4)	1 (1–3)
Clients on first-line regimens ^b^	117 (91%)	124 (95%)	121 (92%)	120 (93%)	111 (83%)	593 (91%)
Clients on second-line regimens ^b^	11 (9%)	7 (5%)	11 (8%)	9 (7%)	22 (17%)	60 (9%)
Baseline CD4 count, cells/µl (median, IQR)^d^ Unrecorded CD4 count	310 (199–430) 19 (15%)	221 (128–353) 16 (12%)	210 (143–328) 6 (5%)	433 (250–629) 39 (30%) ^a^	234 (118–349) 3 (2%)	272 (152–414) 84 (13%)
**Outcomes at 12 months**						
Retained in care	126 (98%)	127 (97%)	130 (98%)	120 (93%)	133 (100%)	636 (97%)
Unsuppressed (viral load > 1000 copies/ml)	12 (9.4%)	4 (3.1%)	3 (2.3%)	6 (4.7%)	4 (3%)	29 (4.5%)
Suppressed (viral load < 1000 copies/ml)	110 (86%)	125 (95%)	123 (93%)	115 (89%)	118 (89%)	591 (91%)
Unknown viral status	6 (5%)	2 (2%)	1 (1%)	8 (6%)	11 (9%)	28 (4.3%)
**Outcomes at 24 months**						
Retained in care^e^	122 (97%)	127 (98%)	132 (100%)	110 (96%)	131 (99%)	622 (98%)
Unsuppressed viral load (> 1000 copies/ml)	10 (7.9%)	2 (1.5%)	3 (2.3%)	4 (3.5%)	3 (3%)	22 (3.5%)
Suppressed viral load (< 1000 copies/ml)	111 (88%)	117 (90%)	121 (92%)	108 (94%)	119 (90%)	576 (91%)
Unknown viral status	5 (4%)	11 (9%)	8 (7%)	3 (3%)	10 (8%)	37 (6%)

^a^ Sample characteristics and 12-month outcomes are for the FBG1 cohort; 24 month outcomes are for the FBG2 cohort. The characteristics of persons in each FBG sample were very similar. In the FBG2 sample, all clients had a recorded baseline CD4 count, while in FBG1 30% did not

^b^ Age, duration on ART, duration on DSDM and regimen are measured at the time of enrollment in the study (January 2017)

^c^ For FBIM, the duration on DSDM is equivalent to the duration on ART. Some clients switched from their DSDM back to FBIM when becoming unsuppressed, but they were retained in their original models for purposes of analysis

^d^ Baseline CD4 count at time of ART initiation. Data missing for < 10% of clients in all models

^e^ Not retained in care (in 2nd year period) included one death (FBIM); the rest were lost to follow-up (LTFU).

### Resource utilization

#### Antiretroviral medications and laboratory tests

A range of ARV formulations were prescribed and dispensed to our study participants. The most common at 24 months were TDF-3TC-EFV for first line therapy, which accounted for 50% of first line formulations, and TDF-3TC-ATV/r for second line therapy, accounting for 27% of second line regimens (Supporting information S3 Table). Dolutegravir (DTG) became available in 2018; 8.2% of clients had switched to DTG formulations by the 24th study month. Clients received an average of 1–2 months of ARVs at a time—there was little adoption of multi-month dispensing during the study periods. The annual MPR was high, with some clients receiving more than 365 days of ARVs over the year.

Viral load testing appeared consistent with guidelines: study participants received an average of one viral load test per year, and with only minor variation by model (Table 3). There was a reduction in other laboratory investigations between the study periods, from 0.62 tests per client in 0–12 months to 0.28 tests per client in 13–24 months (refer to Supporting information S4 Table).

#### Frequency of facility visits and DSDM events

Clients visited healthcare facilities during the study period either for a scheduled (routine) appointment to collect their ARVs (individual collection, group collection, or fast-track drug refill) or for unscheduled visits for HIV-related illnesses, opportunistic illnesses, or other comorbidities (Table 3). In addition to facility visits, the CCLAD, CDDP, and FBG models held DSDM-specific events, or interactions, such as community-based clinical/TB assessments, group viral load sessions, ARV collections, and adherence support meetings (Supporting information S5-S6 Tables). The available data indicated a reduction of almost half (48%) in the total recorded DSDM events between the two study periods, which may reflect actual changes in clients’ participation, deterioration in record keeping, or both. Actual implementation of the DSDM models differed slightly from MOH guidelines (Kiggundu 2020) in the frequency of facility ART visits, DSDM interactions, and viral load tests. These differences diminished over the two-year study period, as greater standardization occurred in DSDM implementation.

**Table 3:** Resource utilization per client, by model and study period (mean per client per annum, total cohort)

Service/item (Mean frequency per client/annum)			FBIM (n = 128)		CCLAD (n = 131)			CDDP (n = 132)			FBG1/2 (n = 129, 115)^a^			FDR (n = 133)	Total (n = 653)			
**Months 0–12**																		
**Laboratory investigations**																		
Viral load tests^b^ (mean number per client)			1.05		1.15			1.18			1.09			1.02	1.09			
Clients with unrecorded viral status			6 (5%)		2 (2%)			1 (1%)			8 (6%)			11 (9%)	28 (4.3%)			
All other (non-VL) tests			0.38		0.84			0.24			1.19			0.44	0.62			
**Facility visits / DSDM events**																		
Mean duration of dispensing interval (months)			1.6		1.9			1.7			1.6			2.0	1.7			
Facility visits^c^			7.74		6.44			6.91			7.57			6.12	6.95			
DSDM events^d^			0.00		3.60			4.16			6.12^e^			0.00	2.77			
Total interactions			7.74		10.04			11.07			13.69			6.12	9.72			
**Months 13–24**																		
**Laboratory investigations**																		
Viral load tests^b^ (mean number per client)			0.98		0.83			0.92			1.15			0.92	0.95			
Clients with unrecorded viral status			5 (4%)		11 (9%)			8 (7%)			3 (3%)			10 (8%)	37 (6%)			
All other (non-VL) tests			0.29		0.42			0.05			0.54			0.15	0.28			
**Facility visits / DSDM events**																		
Mean duration of dispensing interval (months)			1.6		2.0			2.0			1.3			2.1	1.8			
Facility visits^c^			7.63		5.92			6.07			9.05			5.82	6.84			
DSDM events^d^			0.00		2.00			1.92			6.6 ^e^			0.00	2.01			
Total interactions			7.63		7.92			7.99			15.70			5.82	8.85			

^a^ Months 0–12 are FBG1 data. Months 13–24 are FBG2 data

^b^ Viral load test frequency is guided by the MOH Treatment Guidelines. Other tests are done if clinically indicated

^c^ Facility visits could be either scheduled ARV collections or unscheduled visits for other needs. Included ARV collections for all DSDM clients, even if collected by a community/group member on behalf of the client – and costs of pharmacy and nurse time were split between group members

^d^ DSDM events count excluded the ARV pickups from facilities which were counted as facility visits

^e^ FBG support groups could occur in community or at facilities, but are all labelled here as DSDM events

#### Total cost per client and cost per outcome

Unit costs of the resources utilized by participating clients are available in Supporting information S1, S3-S6 Tables). For the second study period, which may better reflect costs going forward, the annual mean cost per client in care was $141, $146, $150, $152 and $166 for the FBG, CDDP, CCLAD, FBIM (standard of care) and FDR models, respectively (Table 4). FBIM and FDR costs were largely driven by having greater proportions of clients on second-line regimens (9% and 17% respectively, by the end of the study period). The mean annual cost per second-line (SL) client across all models was more than double that of first-line (FL) client ($135 FL vs. $343 SL). Due to there being very low lost-to-follow-up (2%) within the study cohort, the cost per client *not* retained in care is not presented. However, the mean cost per virally suppressed client (at 24 months after study enrolment) was $150, $158, $167, $173 and $184, for FBG, CDDP, CCLAD, FBIM and FDR respectively, a 10% increase from the mean cost per client in care (averaged across all models).

ARVs and laboratory tests were the main cost drivers for all models – 74% and 9% respectively of total costs (Table 4) - followed by the prevention and treatment of opportunistic infections which included Isoniazid and Pyridoxine (8% on average). If these three cost components are removed from the totals, the mean annual service delivery cost per client in care was $10 for FDR, $12 CDDP, $14 FBIM, $16 FBG, and $16 for CCLAD. Human resource costs for facility visits (3% on average) varied across the models, based on the different staff involved, their salary scales, and the length of time and frequency of each interaction. Participants in the FBGs (pregnant and breastfeeding women) appeared to have a greater proportion of personnel costs, due to more frequent facility visits and interactions. Human resource costs for the DSDM events/interactions were low (0.1%) because most were group events for which staff costs were shared among the group participants. Site overhead costs (3%) and above-site costs (3%), for supervision, training, management, and implementing partners’ headquarter costs, varied between models but generally account for only a small share of the total per client in care. CCLAD had slightly higher above-site costs than the other models, in part due to their greater supervision, monitoring and headquarters’ operating costs, while CDDP and FDRs had the lowest above-site costs (Table 4).

**Table 4:** Annual average cost per client by cost component, by model and period (US$ 2018)

Mean cost per enrolled client per annum (US$)	FBIM		CCLAD		CDDP		FBG		FDR		Overall	
	***0-12 m***	***13-24 m***	***0-12 m***	***13-24 m***	***0-12 m***	***13-24 m***	***FBG1***	***FBG2***	***0-12 m***	***13-24 m***	***0-12 m***	***13-24 m***
	***(n = 128)***	***(n = 126)***	***(n = 131)***	***(n = 130)***	***(n = 132)***	***(n = 132)***	***(n = 129)***	***(n = 115)***	***(n = 133)***	***(n = 132)***	***(n = 653)***	***(n = 635)***
ARVs (including SCM^a^ costs)	108.31	115.33	104.48	103.20	116.89	112.76	104.75	96.88	136.83	133.96	114.38	112.84
Non-ARV meds (including SCM costs)	20.17	9.99	16.71	20.10	22.17	10.12	22.07	13.13	23.44	11.10	20.92	12.89
Laboratory tests	14.10	13.04	14.99	11.21	14.96	11.40	14.75	14.85	13.84	11.75	14.52	12.38
Facility visits (HR costs^b^)	6.27	5.00	4.09	2.55	2.02	1.47	11.26	6.90	6.22	4.77	5.95	4.06
DSDM events (HR costs)	^c^	^c^	0.29	0.17	0.31	0.17	0.16	0.06	^c^	^c^	0.15	0.08
Site-level costs: transport, overheads	4.50	4.50	7.40	7.40	7.86	7.86	4.51	4.22	2.10	2.10	5.27	5.24
Above-site costs: supervision, training, materials, management	4.58	4.62	5.44	5.44	2.65	2.65	5.28	5.24	2.77	2.79	4.13	4.11
DSDM cost ($) per enrolled	157.93	152.49	153.39	150.07	166.85	146.42	162.77	141.29	185.20	166.48	165.33	151.61
client per year (mean, SD)	(62.51)	(72.04)	(57.78)	(54.94)	(82.34)	(59.52)	(79.53)	(33.7)	(104.47)	(82.51)	(79.76)	(63.65)
Service delivery costs (excl. ARVs, labs & non-ARV meds)	15.35	14.12	17.22	15.56	12.84	12.14	21.20	16.43	11.09	9.66	15.51	13.50

Note these costs relate to the entire cohort, that is, those clients in care at the beginning of each annual study period

^a^ SCM = supply chain management

^b^ Facility visits included human resource costs for ARV refills, fast-track refills, clinical assessments, TB assessments, counselling, drawing blood for lab tests, and unscheduled visits

^c^ FBIM and FDR did not have any community-based DSDM events

## Discussion

By 2018, Uganda had developed and implemented five differentiated models of ART service delivery, including the standard of care, known as facility-based individual management (FBIM). In this two-year observational study, we found that, on average, all five DSDMs achieved good outcomes and cost the provider (Ministry of Health or NGO implementers) an average of $152 per year per client in care. Retention in care averaged a high 98% for the sample as a whole, with a tight range of 96–100%. Viral suppression, which averaged 91%, varied between a low of 88% among clients in FBIM, which served as the primary model for treating complex clients, and a high of 94% among FBG clients. These findings will be valuable to the Ugandan Government and implementing partners in planning for the resourcing, implementation and scale-up of effective DSDMs throughout the country. Our outcomes are consistent with other reports on the CDDP [6, 7] and CCLAD [5] models in Uganda. Similarly, in other African countries, a recent systematic review concluded that retention in care and viral suppression are roughly equivalent to those in conventional models of care [8].

Differences among the models’ costs were explained largely by clients’ ARV regimens and the costs of prevention and treatment of opportunistic infections and other co-morbidities. Service delivery costs, excluding ARVs, laboratory tests and other non-ARV medicines, were modest, ranging from $10-$16 per client, with CCLADs being slightly higher due higher above-site costs while FBGs personnel costs were higher due to increased facility visits and interactions. Although there may not be much room for “savings” to the healthcare system through the new Ugandan DSDMs, their client-centred approach and potential savings in time and convenience for clients are important but were not estimated in this study. Our findings of healthcare system costs are similar to some other recent studies but not with others. A recent observational evaluation in Zambia, for example, found that the standard of care model was less expensive than community-based ART delivery [14]. In South Africa, in contrast, a study of adherence clubs where lower cadre staff (compared to the facility-based standard of care staff) dispensed ARVs to 25–30 members at club meetings found them cost-saving compared with the standard of care [15]. Evaluations of models implemented in cluster-randomized trials that explicitly emphasized multi-month dispensing of ARVs have also observed modest cost savings [16]. We note that in Uganda, over the period of this study, participants made more facility ARV refill visits (scheduled) for medication collection than called for in guidelines. Since the study ended, Uganda has implemented longer dispensing intervals for ARVs, which may lead to lower costs for models that are able to dispense six-month supplies to a large share of clients. Numerous studies have also found that DSDMs do substantially reduce costs to clients, primarily for transport and time [17]. With equivalent or better outcomes and large benefits to clients, the finding that differentiated models do not greatly reduce provider costs does not diminish their societal value, and the data presented here will enable the Ugandan Government and partners to consider the optimal mix of DSDMs to meet client needs, their resource requirements and plan for their scale-up.

Our study applied the a cost-outcome analysis approach, using a retrospective micro-costing based on clients’ records. While the findings in the paper are specific to Uganda (and to the models in use during the study period), the approach and methods are widely generalizable. The methodology could readily be replicated in other settings and would provide value insights into patients’ progression in DSDMs over time. The study had a number of limitations, however, largely stemming from our reliance on routinely-collected, retrospective data. Because of incomplete electronic client medical records at some sites, we relied on individual clients’ paper ART Care Cards, which are removed from healthcare facilities when clients die. As a result, we likely undercounted deaths in the 0–12 month sample, and during the second study period, we identified only one death. Our outcomes measures were therefore limited to clients surviving at 24 months, possibly causing us to overstate rates of retention and viral suppression. Additionally, clients can only join the DSDMs (except FBIM) when considered as established on treatment (stable), which includes having a suppressed viral load test result. All “unestablished” or higher risk patients remain in FBIM. We would therefore expect FBIM to have lower viral suppression rates than the other models, as we saw in this study. We also struggled with incomplete records of DSDM interactions, as model registers were poorly maintained and this worsened in the second study period. The decrease in DSDM interactions, from an annual average 2.85 in the first period to 2.05 in the second period, may thus reflect either an actual reduction in DSDM interactions or a worsening in record-keeping between the years. We could not include children and adolescents in our sample, but their treatment outcomes in DSDMs should be studied further. Finally, estimates of staff time spent for each type of event were obtained through interviews with staff. Self-reported time use may not be accurate, and we excluded non-client-facing activities such as record keeping, stock management, and breaks. We thus may have underestimated these human resource costs for every model. In a separate facility-level analysis of total salary costs/client, we estimated an additional personnel cost of $2.20 per client per year, for these non-client-facing activities. These could be added to the totals for each model in Table 4.

**In conclusion**, differentiated ART service delivery in Uganda achieved excellent treatment outcomes at a cost similar to standard of care (FBIM). While large budgetary savings might not be immediately realized, the reallocation of “saved” staff time due to multi-month dispensing and reduced facility visits could improve health system efficiency as facilities and clients gain more experience with the DSD models. These findings support the scale-up of the DSDMs so as to provide an optimal package of client-centred options to meet the range of client situations and needs. Future research could explore the savings to clients, their increased convenience, adherence and treatment success over longer periods of time.

## Electronic supplementary material

Below is the link to the electronic supplementary material.

S1 Table. Unit costs for human resources for services, laboratory tests and ARV formulations.
S2 Table. Methods for cost estimation by cost category.
S3 Table. DSDM patient’s ARV formulations (as at the end of 24-month study period).
S4 Table. Types and frequency of diagnostic tests performed.
S5 Table. Types and frequency of facility-based services.
S6 Table. Types and frequency of DSDM events (non-facility based).
S1 File. Sampling: Selection of sites.

## Data Availability

All resource utilization and costing data underlying this article are incorporated into the article and its online supplementary material. The client level data that support the findings of this study are owned by the Ugandan Ministry of Health, and so are not publicly available. Data are however available from the authors upon reasonable request and with permission of the Ministry of Health (Uganda).

## Abbreviations

CCLAD: Community client-led ART delivery.
CDDP: Community drug distribution pointsr.
FBG: Facility-based groups.
FBIM: Facility-based individual management.
FDR: Fast-track drug refills.
IAC: Intensive adherence counselling.
LTFU: Lost to follow-up.
MMS: Multi-month scripts.
MPR: Medication possession rate.
PLW: Pregnant and lactating women.
VL: Viral load.
VS: Virally suppressed.
