# Supplementary material for: Similar costs and outcomes for differentiated service delivery models for HIV treatment in Uganda
Source: BMC Health Serv Res. 2022 Nov 3;22:1315. doi: 10.1186/s12913-022-08629-4 (PMC9635081; doi:10.1186/s12913-022-08629-4)
Supplement: Supplementary file 1 — S1 Table. Unit costs for human resources for services, laboratory tests and ARV formulations. S2 Table. Methods for cost estimation by cost category. S3 Table. DSDM patient’s ARV formulations (as at the end of 24-month study period). S4 Table. Types and frequency of diagnostic tests performed. S5 Table. Types and frequency of facility-based services. S6 Table. Types and frequency of DSDM events (non-facility based). S1 File. Sampling: Selection of sites. [file 12913_2022_8629_MOESM1_ESM.pdf]

SUPPLEMENTARY DATA

Similar Costs and Outcomes for Differentiated Service Delivery Models for HIV Treatment in Uganda

Corresponding author:
Teresa Guthrie
ORCID iD: 0000-0001-9542-6693
Health Economics and Epidemiology Research Office, Department of Internal Medicine, School of Clinical Medicine, Faculty of Health Sciences, University of the Witwatersrand, Johannesburg, South Africa
E-mail: guthriehealthfinancingconsult@gmail.com

Supplementary Data

S1 Table. Unit costs for human resources for services, laboratory tests and ARV formulations

Service / event / item	Mean cost (US$ 2018)
**Human resource costs for facility-based services**	**Per service**
Drug refill	0.09
Fast-track drug refill	0.19
Comprehensive clinical assessment	1.21
TB assessment at clinic	1.52
Unscheduled clinic visit for OIs, co-morbidities, other	1.71
Drawing blood at clinic	0.23
FBG quick clinical assessment	0.09
Counselling: education on basic HIV prevention and disclosure	1.52
Counselling: ART preparation, initiation, support, monitoring	1.40
Counselling: Progression on treatment	1.34
Intensive adherence counselling	2.60
**Human resource costs for community-based services**	**Per service**
CCLAD pre-med/meds collection/support meetings*	- *
Viral Load testing meeting (CCLAD & CDDP)**	0.11
Counselling session (CCLAD & CDDP)**	0.04
Clinical assessment (CCLAD & CDDP)**	0.09
TB assessment (CCLAD & CDDP)**	0.09
CDDP TB Case Finding Guide**	0.04
CDDP Drug Pick Up at community**	0.04
*Volunteer time only, not valued.<br>** Personnel time for group events, split between all patients in the group.	
**Laboratory test**	**Per test**
Viral Load	12.30
CD4	9.60
TPHA (Syphilis test)	2.10
Complete Blood Count (CBC)	1.20
Malaria RDT	0.80
Serum Crag	4.10
GeneXpert	11.80
Haemoglobin	1.00
Urinalysis	0.80
HCG (Pregnancy test)	0.30
**First-line ARV regimens (fixed-dose combinations)**	**12 month supply*** **
AZT/3TC/NVP	73.61
TDF/3TC/EFV	76.04
TDF/3TC/NVP	77.26
TDF/3TC/DTG	99.16
AZT/3TC/EFV	100.38
ABC/3TC/EFV	114.37
ABC/3TC/DTG	124.71
**Second-line regimens**	**12 month supply*** **
ABC/3TC/NVP	102.81

AZT/3TC/DTG	110.72
TDF/3TC/ATV/r	231.78
AZT/3TC/ATV/r	243.33
ABC/3TC/ATV/r	257.33
TDF/3TC/LPV/r	274.48
AZT/3TC/LPV/r	286.04
ABC/3TC/LPV/r	300.03

***Price includes supply chain management costs of 24.73%.

S2 Table. Methods for cost estimation by cost category

Resource Input	Quantification method (Q)	Valuation technique (P)
**Variable costs (direct-patient resources reported in patient medical records and DSDM registers)**		
Medicines	Prescribed medicines during the study period, including ARVs and non ARVs.	The US$ (2018) unit price of medicines were used, as provided by the MOH Quantification Procurement & Purchasing Unit (QPPU). Total cost of drugs was estimated as quantity multiplied by unit cost.
Supply chain management costs	All medicines prescribed during the study period had a supply management cost applied to them.	The actual supply chain management cost varied depending on the medicine. An average mark-up of 24.725% on drug costs across all medicines was applied at the recommendation of the QPPU.
Viral load and other laboratory tests	Number of viral load (VL) tests and other laboratory tests done during the study period.	Unit price of VL test and others (US$2018) provided by the Central Public Health Laboratories (CPHL), and multiplied by the number of tests done over the period.
Human resources	The frequency of facility services or DSDM community events attributed to each patient were obtained from their ART care cards and the DSDM registers.<br>The different cadre engaged in providing specific types of facility services and DSDM events, and the average amount of time they each spent on these, were obtained through staff interviews – specific to each site and model-type. For group/community events, the average number of patients per group/ event were also obtained through staff interviews.	Public staff salaries were obtained from the public salary scales for 2017 and 2018. IP's staff annual gross salaries (full cost to company) were obtained from their expenditure records. The cost per minute per cadre was calculated and applied to the reported amount of time (minutes) spent on facility services and DSDM community events. The HR cost per service/ event was multiplied by the numbers of each used by each patient. For group/ community events, the HR cost was divided by the average number of patients in the group/ event, and attributed to individual sampled patients.<br>A sensitivity analysis was performed to assess the possible range of under/over-reporting by staff.
**Fixed costs (resources used for the facility and DSDM operation – both site-level and above-site)**		
Per diem and travel allowances for expert patient and staff	The number of per diems per model was based on the length of stay away from the facility, obtained through interviews with programme staff and volunteers.	The Government per diem rate (2018) and transport rate was applied to the number of support visits.
Vehicle maintenance costs	Through staff interviews the numbers of vehicles that were used in the DSDM service delivery were identified, and % of their maintenance costs that should be apportioned to DSDM activities was obtained.	Actual maintenance costs were obtained from the IP expenditure records, and the % attributed to the DSDM. By step down costing approach, these were then attributed to each patient in the DSDM.
Printing and record keeping	Total stationary, printing and files consumed in each year and their costs were based on actual expenditure. Through staff interviews the relevant % that should be apportioned to DSDM activities was obtained.	A proportion of the actual annual expenditure was applied for each specific model based on staff interviews, and then attributed to each patient in the DSDM (step down allocation).
Training costs	These included venue hire, per diem, travel costs, meals and refreshments and the development and supply of all training materials. Through staff interviews the relevant % that should be apportioned to specific sites and models.	Valuation was based on actual expenditure by the selected site or implementing partner (IP).<br>If training was a once-off cost before establishing the models, these costs were considered as capital investment and were discounted and annualized over a 3-year period.
Materials for repackaging of drugs	Actual quantities of the different materials used for repackaging of drugs were obtained through review of records. Through staff interviews the	Quantities of different materials multiplied by their unit prices, obtained from the IP expenditure records.

Resource Input	Quantification method (Q)	Valuation technique (P)
	relevant % that should be apportioned to DSDM activities was obtained.	
Communication costs	Actual expenditure on communication that was relevant to DSDMs.	Where necessary, communication expenditure was apportioned to specific sites and models (if the expenditure was not separately kept for these models).
Overheads/ utilities	Actual expenditure on overheads for the site from expenditure records.<br>Through staff interviews the relevant % that should be apportioned to DSDM activities was obtained.	Apportioned total overheads expenditure to DSDM using the relevant %.
Facility and DSDM programme management (PM) & administration costs, incl. oversight and supervision	Actual expenditure on programme managers/ admin staff from expenditure records.<br>Through staff interviews the relevant % that should be apportioned to DSDM activities was obtained.	Apportioned total PM costs expenditure to DSDM using the relevant %.<br>A proportion of all these shared costs was then allocated to each DSDM patient using an allocation factor, per site and model, to obtain an average indirect cost per patient. This allocation factor was calculated as the numbers of ART visits per annum / total number of out-patient visits per annum at the facility.
Materials and supplies	Actual quantities of the different materials used for DSDM start up and for on-going operations.	Quantities of different materials multiplied by their unit prices (from IP expenditure records).
IEC materials	Actual expenditure on IEC materials for DSDMs, shared across DSDMs if more than one was being implemented	Quantities of different materials multiplied by their unit prices (from IP expenditure records).
Community sensitization and mobilization	Actual expenditure on mobilization costs per site from records. Through staff interviews the relevant % that should be apportioned to DSDM activities was obtained.	Apportioned total IP expenditure to DSDM using the relevant %.
**Capital Costs**		
Vehicles / Motorcycles / Bicycles	Quantification of vehicles/motorcycles/bicycles were done through the KIIs, and their share of use for the specific models estimated (if not fully utilized by the model).	Current replacement costs for vehicles was obtained from MOH procurement price list. The estimated annual cost was annualized value of the vehicles.
Buildings	Space consumed by DSDM activities was measured in square meters.	A square meter was valued at UGX 61,240 (discounted and annualized), which was provided by the MOH (budget framework).

S3 Table. DSDM patient's ARV formulations (as at the end of 24-month study period)

ARV formulation	CCLAD (n=130	CDDP (n=132	FBG (Gp2) (n=115)	FBIM (n=126	FDR (n=132	Total (n=635
**First-line regimens**	95%	92%	99%	90%	84%	92%
AZT/3TC/NVP	*28%*	*32%*	*12%*	*18%*	*21%*	*22.7%*
TDF/3TC/EFV	*35%*	*25%*	*82%*	*60%*	*32%*	*45.8%*
TDF/3TC/NVP	*13%*	*8%*	*4%*	*1%*	*12%*	*7.9%*
TDF/3TC/DTG	*12%*	*17%*	*1%*	*1%*	*8%*	*8.0%*
AZT/3TC/EFV	*5%*	*10%*	*0%*	*10%*	*11%*	*7.4%*
AZT/3TC/DTG	*1%*	*0%*	*0%*	*0%*	*0%*	*0.2%*
ABC/3TC/EFV	*1%*	*0%*	*0%*	*0%*	*0%*	*0.2%*
**Second-line regimens**	5%	8%	1%	10%	16%	8%
TDF/3TC/ATV/r	*1%*	*2%*	*1%*	*4%*	*3%*	*2.2%*
AZT/3TC/ATV/r	*0%*	*1%*	*0%*	*2%*	*2%*	*0.8%*
ABC/3TC/ATV/r	*2%*	*1%*	*0%*	*0%*	*6%*	*1.7%*
TDF/3TC/LPV/r	*2%*	*2%*	*0%*	*3%*	*3%*	*2.0%*
ABC/3TC/LPV/r	*0%*	*0%*	*0%*	*0%*	*2%*	*0.3%*
AZT/3TC/LPV/r	*1%*	*2%*	*0%*	*1%*	*1%*	*0.8%*

S4 Table. Types and frequency of diagnostic tests performed

Test/client/annum	CCLAD (n=131)	CDDP (n=132)	FBG1 (n=129)	FBIM (n=128)	FDR (n=133)	Total (n=653)
**Months 0-12**						
Viral load*	1.15	1.18	1.09	1.05	1.02	1.09
Haemoglobin	0.34	0.05	0.21	0.01	0.03	0.13
CD4 count	0	0	0.02	0.05	0.11	0.04
TB sputum	0.01	0.02	0.05	0.03	0.05	0.03
All other tests	0.5	0.17	0.91	0.29	0.26	0.43
Total tests/client	1.98	1.42	2.27	1.43	1.46	1.71
Total non-VL tests/client	0.84	0.24	1.19	0.38	0.44	0.62
**Months 13-24**			**FBG2 (n=115)**			
Viral load*	0.83	0.92	1.15	0.98	0.92	0.95
Haemoglobin	0.13	0	0.2	0.02	0.01	0.07
CD4 count	0.04	0	0	0.04	0.01	0.02
GeneXpert **	0.01	0.01	0.01	0.02	0.02	0.01
All other tests	0.25	0.05	0.33	0.2	0.11	0.18
Total tests/client	1.25	0.97	1.69	1.26	1.07	1.24
Total non-VL tests/client	0.42	0.05	0.54	0.29	0.15	0.28
% change in frequency of all tests between periods	-37%	-32%	-26%	-12%	-27%	-27%

S5 Table. Types and frequency of facility-based services

Facility services (0-12mths)	CCLAD *(n=131)*	CDDP *(n=132)*	FBG *(n=129)*	FBIM *(n=128)*	FDR *(n=133)*	Overall *(n=653)*
Retained in care (RIC), n (%)	*127 (96.9%)*	*130 (98.5%)*	*120 (93%)*	*126 (98.4%)*	*133 (100%)*	*636 (97.4%)*
**Facility services, n (av/RIC client/ann)**						
ART refill visits	837 (6.59)	910 (7)*	977 (8.14)	987 (7.83)	813 (6.11)	4524 (7.11)
Comprehensive clinical assessment (1/2/3/6/9 mths or annual assessment)	117 (0.89)	121 (0.92)	624 (4.84)	817 (6.38)	162 (1.22)	1841 (2.82)
Fast-track drug refill assessment	6 (0.05)	1 (0.01)	10 (0.08)	0 (0)	488 (3.67)	505 (0.77)
TB assessment at clinic	23 (0.18)	28 (0.21)	237 (1.84)	224 (1.75)	139 (1.05)	651 (1)
Unscheduled clinic visit/s for HIV-related illnesses, OI, co-morbidities	6 (0.05)	2 (0.02)	0 (0)	4 (0.03)	1 (0.01)	13 (0.02)
** ARV refills for CDDP clients occurred at non-facility pick-up points, but are included here for comparison of numbers of refills across DSDMs*						
**Counselling session (n, av/RIC client/ann)**	**73 (0.56)**	**28 (0.21)**	**388 (3.01)**	**174 (1.36)**	**210 (1.58)**	**873 (1.34)**
Education on basic HIV prevention and	19 (0.15)	5 (0.04)	257 (1.99)	64 (0.5)	120 (0.9)	465 (0.71)
Progression on Rx counselling	8 (0.06)	2 (0.02)	49 (0.38)	24 (0.19)	16 (0.12)	99 (0.15)
ART preparation, initiation, support,	34 (0.26)	5 (0.04)	56 (0.43)	15 (0.12)	46 (0.35)	156 (0.24)
Home based care counselling	5 (0.04)	0 (0)	1 (0.01)	1 (0.01)	5 (0.04)	12 (0.02)
Intensive adherence counselling	7 (0.05)	16 (0.12)	25 (0.19)	70 (0.55)	23 (0.17)	141 (0.22)

S6 Table. Types and frequency of DSDM events (non-facility based)

DSDM Events (0-12mths)	CCLAD *(n=131)*		CDDP *(n=132)*		FBG *(n=129)*		Total (CCLAD, CDDP, FBG) *(N=392)*	
**DSDM event (n, av/client/ann)**	**472**	***3.6***	**1320**	***10.0***	**789**	***6.1***	**2581**	***6.6***
CCLAD Viral Load testing meeting	12	0.09	n/a	n/a	n/a	n/a	12	0.03
CCLAD counselling session (in community)	100	0.76	n/a	n/a	n/a	n/a	100	0.26
CCLAD clinical assessment (in community)	125	0.95	n/a	n/a	n/a	n/a	125	0.32
CCLAD TB assessment (in community)	235	1.79	n/a	n/a	n/a	n/a	235	0.60
CDDP clinical assessment (in community)	n/a	n/a	229	1.73	n/a	n/a	229	0.58
CDDP counselling session (in community)	n/a	n/a	289	2.19	n/a	n/a	289	0.74
CDDP TB Case Finding Guide	n/a	n/a	2	0.02	n/a	n/a	2	0.01
CDDP TB community assessment	n/a	n/a	28	0.21	n/a	n/a	28	0.07
CDDP drug pick up (in community) (=ARV refills)	n/a	n/a	771	5.84	n/a	n/a	771	1.97
FBG Group meetings	n/a	n/a	n/a	n/a	627	4.75	627	1.60
FBG Quick clinical assessment	n/a	n/a	n/a	n/a	161	1.22	161	0.41
Home based care / palliative care / symptom management at home	n/a	n/a	1	0.01	1	0.01	2	0.01

DSDM Events (13-24mths)	CCLAD (n=130)		CDDP (n=132)		FBG (n=115)		Total (CCLAD, CDDP, FBG) (N=377)	
**DSDM event (n, av/client/annum)**	**260**	***2.0***	**253**	***1.9***	**765**	***6.7***	**1278**	***3.4***
CCLAD Viral Load testing meeting	0	-	n/a	n/a	n/a	n/a	0	-
CCLAD counselling session (in community)	23	0.18	n/a	n/a	n/a	n/a	23	0.06
CCLAD clinical assessment (in community)	71	0.55	n/a	n/a	n/a	n/a	71	0.19
CCLAD TB assessment (in community)	161	1.24	n/a	n/a	n/a	n/a	161	0.43
CDDP clinical assessment (in community)	n/a	n/a	199	1.51	n/a	n/a	199	0.53
CDDP counselling session (in community)	n/a	n/a	5	0.04	n/a	n/a	5	0.01
CDDP TB Case Finding Guide	n/a	n/a	3	0.02	n/a	n/a	3	0.01
CDDP TB community assessment	n/a	n/a	44	0.33	n/a	n/a	44	0.12
CDDP drug pick up (in community) (=ARV refills)	n/a	n/a	0	-	n/a	n/a	0	-
FBG Group meetings	n/a	n/a	n/a	n/a	764	5.79	764	2.03
FBG Quick clinical assessment	n/a	n/a	n/a	n/a	0	-	0	-
Home based care / palliative care / symptom management at home	n/a	n/a	1	0.01	1	0.01	2	0.01

n/a = not applicable - where that particular model does not have those particular types of events.

S1 File. Sampling: Selection of sites

The study population for Phase 1 (top-down collection of implementers' operational costs) were all the DSDMs in Uganda that would have been in operation longer than 6 months (by October 2017, the data collection point). There were 783 health facilities/sites that were implementing DSDMs in Uganda, serving a total number of 175,000 clients (according to IP reports).

Multi-stage purposive sampling was applied, since a randomized representative sample was beyond the time and resources available for this rapid study.

The DSDM site's operating length of time was considered: only sites that had been in operation for more than 6 months were included in the sampling frame. In addition, the size of the DSDM sites, in terms of the numbers of clients served by each model, was considered. Those that were defined as too large or too small (dependent upon the model type) were excluded. Table 1 shows the different DSDMs' average numbers of clients, and indicates the exclusion rules applied for each. Models that were outliers (i.e. very small or very large) were excluded, as determined below.

Sampling: Table 1. Definition of DSDM sizes (based on numbers of clients by type of DSDM) and exclusion criteria

	CCLAD	CDDP	FBG	FDR
Average # clients per group or site	8	33	49	685
Rule for exclusion: min # of clients	<4	<10	<10	<100
Rule for exclusion: max # of clients	>12	>400	>100	>1000
# sites excluded from sampling frame	0	49	64	64

After these exclusions, 605 eligible sites remained in the sampling frame for inclusion. From these, 47 were selected through stratified purposive sampling.

The eligible models were first stratified by the type of model, and approximately 7%-8% from the CCLADs, CDDPs, FBGs and FDRs were selected. For the FBGs, additional effort was made to include their different client groups (pregnant and lactating women, children and adolescents, families). Only 4 FBIMs sites were added (at the request of the MOH), so as to include those clients who did not, or could not, join the other DSDMs, either by choice or by virtue of being complex (according to the Guidelines).

Within the model-types, the sites were then clustered according to their size (number of clients), and sites selected so as to have more or less similar amounts of each size, as shown in Table 2.

Sampling Table 2. Defining site size by client numbers, and sites selected in each cluster

Sites' number of clients	CCLAD	CDDP	FBG	FDR	Total in sample
**Definition: Small**	4-6	10-30	10-20	100-400	12
**Definition: Medium**	7-9	31-80	21-50	401-700	19
**Definition: Large**	10-12	81-400	51-100	701-1000	12

* NB. The size of the 4 selected FBIM's were not known at the time of sampling, and therefore the clustering by size did not apply to the FBIMs.

Sites were then purposively sampled based on their location, so as to ensure adequate amounts from each of the four Ugandan regions. The most extreme locations and hard to reach sites were omitted due to time and resource constraints. Finally, the selection of the 47 sites was made so as to include 10 different IPs, as shown in Table 3.

Sampling Table 3. Number of sites per IP and per model included in the sample

Service Provider	CCLAD	CDDP	FBG	FDR	FBIM	Total
ASSIST North	2					2
IDI		1	4	1	6	12
MILDMAY UGANDA	2	1	1			4
RHITES-EC	1					1
TASO	2	1	3	1		7
UEC/UCMB		2		3	1	6
UPMB		1	1			2
PIDC-COE-BAYLOR	1			1		2
RHITES-SW			6		2	8
REACH-OUT MBUYA		1		1	1	3
**Total Ph1 sample sites**	**8**	**7**	**15**	**7**	**10**	**47**

* Many of the Baylor-Uganda sites were excluded based on their short period of operation. Therefore, two of their 8 eligible sites were included.
IDI = Infectious Disease Institute]
UCMB = Uganda Catholic Medical Bureau
TASO = The AIDS Support Organization
UPMB = Uganda Protestant Medical Bureau
RHITES- EC = Regional Health Integration to Enhance Services- East and Central
RHITES- SW = Regional Health Integration to Enhance Services – South West

In summary, Table 4 indicates the characteristics of the DSDM sites that were considered in the stratified purposive sampling. Refer to the following Table 5 below for the detailed names of sites and their locations.

Sampling Table 4. Summary characteristics of sites

Site characteristic	Number included in sample	Percentage in sample
**DSDM type:**		
CCLAD	9	19%
CDDP	6	12.5%
FBG	22	47%
FDR	6	12.5%
FBIM	4	9%
**Regional location:**		
North	9	19%
Central	17	36%
East	5*	11%
West	16	34%
**Implementing Partner:**		
ASSIST NORTH	2	4.2%
BAYLOR	2	4.2%
IDI	12	25.5%
MILDMAY	4	8.5%
REACHOUT MBUYA	3	6.4%
RHITES-EC	1	2.1%
RHITES-SW	8	17%
TASO	7	15%
UCMB	6	12.8%
UPMB	2	4.3%
**Facility type:**		
Hospital	12	26%
Health Centre IV	11	23%
Health Centre III	13	28%
TASO sites	6	13%
**Facility ownership:**		
Government	26	55%
Private not for profit (PFNP)	21	45%

* East region had fewer sample sites due to the large number of DSDMs operated there by TASO, who are conducting their own cost-efficiency analysis and therefore did not require as many sites included in this study.

Sampling Table 5. List of 47 sampled sites

Implementing Partner	Region	District	Health Facility	Health facility ownership	Type of DSDM
ASSIST NORTH	NORTH	LIRA	OGUR HC IV	GOVT	CCLAD
ASSIST NORTH	NORTH	OYAM	ANYEKE HC IV	GOVT	CCLAD
BAYLOR	CENTRAL	KAMPALA	BAYLOR HOSPITAL	PNFP	CCLAD
BAYLOR	CENTRAL	KAMPALA	BAYLOR HOSPITAL	PNFP	FDR
IDI	CENTRAL	KAMPALA	KISWA HC III	GOVT	CDDP
IDI	CENTRAL	MASINDI	KYATIRI HC III	GOVT	FBG
IDI	CENTRAL	KAMPALA	KOMAMBOGA HC III	GOVT	FBG
IDI	CENTRAL	KAMPALA	KISUGU HC III	GOVT	FBG
IDI	CENTRAL	KAMPALA	KISWA HC III	GOVT	FBG
IDI	CENTRAL	KIBOGA	LWAMATA HC III	GOVT	FBIM
IDI	WEST	HOIMA	BUHIMBA HC III	GOVT	FBIM
IDI	NORTH	ADJUMANI	ADJUMANI HOSPITAL	GOVT	FBIM
IDI	NORTH	ADJUMANI	DZAIPI HC III	GOVT	FBIM
IDI	WEST	MASINDI	BWIJANGA IV	GOVT	FBIM
IDI	CENTRAL	KAMPALA	KISWA HC III	GOVT	FBIM
IDI	CENTRAL	KAMPALA	IDI MULAGO	PNFP	FDR

MILDMAY	CENTRAL	MITYANA	MITYANA GENERAL HOSPITAL	GOVT	CCLAD
MILDMAY	CENTRAL	WAKISO	MILD MAY HOSPITAL	PNFP	CCLAD
MILDMAY	CENTRAL	WAKISO	KAJJANSI HC IV	GOVT	CDDP
MILDMAY	CENTRAL	WAKISO	KIRA HC III	GOVT	FBG
REACHOUT MBUYA	CENTRAL	KAMPALA	REACHOUT MBUYA	PNFP	CDDP
REACHOUT MBUYA	CENTRAL	LUWERO	St. MARY'S KASALA	PNFP	FBIM
REACHOUT MBUYA	CENTRAL	LUWERO	St. MARY'S KASALA	PNFP	FDR
RHITES -EC	EAST	MAYUGE	KITYERERA HC IV	GOVT	CCLAD
RHITES -SW	WEST	IBANDA	NYAMAREBE HC III	GOVT	FBG
RHITES -SW	WEST	BUSHENYI	BUSHENYI HC IV	GOVT	FBG
RHITES -SW	WEST	IBANDA	RUHOKO HC IV	GOVT	FBG
RHITES -SW	WEST	BUHWEJU	NSIIKA HC IV	GOVT	FBG
RHITES -SW	WEST	MITTOMA	MITOOMA HC IV	GOVT	FBG
RHITES -SW	WEST	SHEEMA	KITAGATA HOSPITAL	GOVT	FBG
RHITES -SW	WEST	ISINGIRO	NYARUBUNGO HC III	GOVT	FBG
RHITES -SW	WEST	IBANDA	NYAMAREBE HC III	GOVT	FBIM
RHITES -SW	WEST	BUSHENYI	BUSHENYI HC IV	GOVT	FBIM
TASO	WEST	MASINDI	TASO	PNFP	CCLAD
TASO	EAST	JINJA	TASO	PNFP	CCLAD
TASO	NORTH	GULU	TASO	PNFP	CDDP
TASO	EAST	TORORO	TASO	PNFP	FBG
TASO	EAST	JINJA	TASO	PNFP	FBG
TASO	EAST	MBALE	TASO	PNFP	FDR
UCMB	WEST	BUSHENYI	St. DANIEL COMBONI HOSPITAL	PNFP	CDDP
UCMB	NORTH	OYAM	POPE JOHN HOSPITAL ABER	PNFP	CDDP
UCMB	NORTH	OYAM	POPE JOHN HOSPITAL ABER	PNFP	FBIM
UCMB	WEST	BUSHENYI	St DANIEL COMBONI HOSPITAL	PNFP	FDR
UCMB	NORTH	OYAM	POPE JOHN HOSPITAL ABER	PNFP	FDR
UCMB	NORTH	GULU	LACOR HOSPITAL	PNFP	FDR
UPMB	WEST	HOIMA	AZUR HC IV	PNFP	CDDP
UPMB	WEST	MBARARA	RUHARO MISSIONARY HOSPITAL	PNFP	FBG

* IDI = Infectious Disease Institute]
* UCMB = Uganda Catholic Medical Bureau
* TASO = The AIDS Support Organization
* UPMB = Uganda Protestant Medical Bureau
* RHITES- EC = Regional Health Integration to Enhance Services- East and Central
* RHITES- SW = Regional Health Integration to Enhance Services – South West

PFNP = Private not for profit

HC= Health Centre

Sampling of the 20 sub-set: selection of sites

From the 47 sites, a sub-set of twenty sites were purposively selected for the collection of direct-patient resource utilization. This was primarily due to resource constraints which prohibited all 47 sites from being included. Four sites for each of the five types of DSDMs, i.e. CCLAD, CDDP, FDR, FBG, and FBIM were included, all of which had been in operation for at least a year by the time of the first study period (months 1-12) data collection and were being implemented by seven IPs.

Sampling Table 6. List of 20 Sampled sites for Phase 2

Implementing partner	Number of sites of each model included	Total

	CCLAD	CDDP	FBG	FBIM	FDR	
PIDC-COE-Baylor					1	1
IDI		1		1		2
MildMay			1	1		2
Reach Out Mbuya				1		1
RHITES – SW			2			2
TASO	4	2	1		1	8
UCMB		1		1	2	4
Total	**4**	**4**	**4**	**4**	**4**	**20**
